# Investigating the Combined Effects of Fascial Distortion Model Manual Therapy and Balance–Strength Training in Individuals with Chronic Ankle Instability

**DOI:** 10.3390/sports12010033

**Published:** 2024-01-18

**Authors:** Amin Mohammadi, Seyed Ehsan Sakhtemani, Lukas Trimmel, Krisztina Petricsevics, Alexandra Makai, Istvan Zsenak, Csaba Melczer, Péter Sándor Tardi

**Affiliations:** 1 Faculty of Health Sciences, Institute of Physiotherapy and Sports Science, University of Pécs, 3 Vörösmarty Str., H-7621 Pecs, Hungary; amin.mohammadi@pte.hu (A.M.); ehsan7813.s@gmail.com (S.E.S.); alexandra.makai@etk.pte.hu (A.M.); istvan.zsenak@etk.pte.hu (I.Z.); csaba.melczer@etk.pte.hu (C.M.); 2 Altgasse 20/14, A-1130 Vienna, Austria; lt@lukas-trimmel.at; 3 Eminent Physiotherapy Center, H-7621 Pecs, Hungary

**Keywords:** chronic ankle instability, fascial distortion model, FDM manual therapy, dynamic balance, static balance, ankle range of motion, balance–strength training, Cumberland ankle instability tool

## Abstract

Background: The Fascial Distortion Model (FDM) is a relatively new manual therapy approach in the field of musculoskeletal physical therapy, and its potential effectiveness in treating chronic ankle instability (CAI) remains unexplored. Methods: A randomized controlled trial with 23 participants was conducted. Patients were randomly assigned to either the FDM + balance–strength training (BST) group (*n* = 8), receiving extra FDM sessions weekly in addition to two sessions of BST, or the BST group (*n* = 7). Healthy controls (*n* = 8) did not receive any treatment and participated only in pre- and post-test measurements. Objective measurements including Y-Balance Test Lower Quarter (YBT-LQ), Flamingo Balance Test (FBT), Weight-Bearing Lunge Test (WBLT), ankle joint range of motion (ROM), and Cumberland Ankle Instability Tool (CAIT) were recorded at baseline and the end of the intervention. The results demonstrated significant differences between the FDM + BST and BST groups for supination ROM (*p* = 0.008) and similarly for WBLT (*p* = 0.041), FBT (*p* = 0.40), YBT-LQ (*p* = 0.023), and CAIT score (*p* = 0.008). Moreover, while both groups demonstrated significant improvement at the post-test compared with their pre-test for plantarflexion and pronation ROM, WBLT, and CAIT score, the FDM + BST group demonstrated significant improvements in supination ROM, FBT, and YBT-LQ. Conclusion: Our study suggests that the addition of FDM concepts to a BST may lead to enhanced improvements in ankle ROM, static and dynamic balance, and self-reported outcomes in individuals with CAI compared to BST.

## 1. Introduction

Acute ankle sprains are the most frequent type of ankle injuries in sport. Among ankle sprains, the lateral ligament complex is the most commonly affected area [1]. Despite the prevalence, a significant number of individuals who experience lateral ankle sprains do not seek proper medical attention [2,3]. Consequently, there is a high incidence of recurrent lateral ankle sprains (>70%) following the initial injury [4], with a substantial percentage of these cases (~40%) progressing to chronic ankle instability (CAI) [2].

CAI is a condition defined as recurring episodes or sensations of the ankle giving way, persistent symptoms including pain, muscular weakness, limited ankle range of motion (ROM), a decline in self-reported functional abilities, and the continued occurrence of ankle sprains lasting for more than one year following the initial injury [5]. CAI may result from either functional instability or mechanical instability or most commonly from a combination of these two disorders [5]. According to Lin and Houtenbos [6], individuals with CAI have weaker isometric eversion, inversion, and plantarflexion than healthy controls, but not dorsiflexion. However, these patients have restricted dorsiflexion ROM. Furthermore, a moderate reduction in static balance in patients with CAI, dynamic balance, impairments in peroneal response time, and deficiencies in eversion strength are all components of the multifaceted disorder known as ankle instability [7]. CAI patients will have a significant improvement in dynamic balance that can be raised by both individual and combinations of patient- and clinician-oriented outcomes [8]. 

Rehabilitation interventions for patients with CAI have primarily focused on resistance and balance training. Research has demonstrated that six weeks of resistance and balance training can increase eversion strength and subjective evaluation. Balance training has also been shown to outperform resistance training in improving dynamic balance and providing pain relief [9]. There is strong evidence that balance training significantly improves instability and dynamic balance as well as health-related quality of life in people with chronic ankle instability [10] and that there are positive impacts of balance training and strength training protocols on the CAI population [11]. In the rehabilitation of patients recovering from chronic lateral ankle sprain (LAS), clinicians are advised to incorporate manual therapy techniques, including graded joint mobilizations, manipulations, and both non-weight-bearing and weight-bearing mobilization with movement. These interventions aim to enhance ankle dorsiflexion, proprioceptive function, and weight-bearing tolerance [12]. Moreover, based on a systematic review, joint mobilization has shown clinical efficacy by producing immediate enhancements in short-term outcomes, including improved dynamic balance and increased range of motion in weight-bearing dorsiflexion [13], and another study revealed that six sessions of manual therapy promoted significant improvements in ankle strength, balance, and performance on functional tests, while a single session of manual therapy did not promote significant improvements in these measurements [14].

It is recognized that fascia is a type of soft tissue that continuously connects in three dimensions to support and shape our bodies. In addition, fibrosis is caused by limited movement and discomfort, which change the internal structure of the muscle such that the fascia hardens and attaches to the surrounding tissues [15]. The Fascial Distortion Model (FDM) method is a relatively new manual therapy approach that has gained popularity in recent years, and it could be used for subjects with chronic ankle instability to improve maximum isometric strength and proprioceptive and dynamic balance [16]. In this study, the aim was to compare the effectiveness of a multi-rehabilitation approach for patients with CAI, including balance–strength (BST) training and its combination with the concept of FDM. Additionally, we will conduct a comparative analysis of the outcomes resulting from these interventions and examine their prospective implications for further research. We hypothesized that the addition of FDM concepts to BST could potentially improve the performance of individuals with CAI, particularly in the domains of static and dynamic balance, as well as ankle joint ROM. This hypothesis is rooted in the recognition of the pivotal role that fascia plays, often underappreciated by physiotherapists, in the rehabilitation of musculoskeletal injuries.

## 2. Materials and Methods

### 2.1. Participants

A total of 108 individuals were screened from a university setting and a total of 30 participants were recruited for the study, including 20 individuals with unilateral CAI and 10 healthy control individuals. All participants provided informed consent, and the study was conducted in accordance with the principles outlined in the Helsinki Declaration. Ethical approval was obtained from the Review Board of the National Center for Public Health in Hungary (Approval No. 14339-6/2023/EÜIG).

CAI individuals who were eligible for the study were randomly assigned into two groups of FDM therapy combined with BST and only BST. However, post-intervention follow-up was only completed with 15 patients and 8 healthy controls. Demographic data for participants are represented in Table 1.

**Figure 1:**
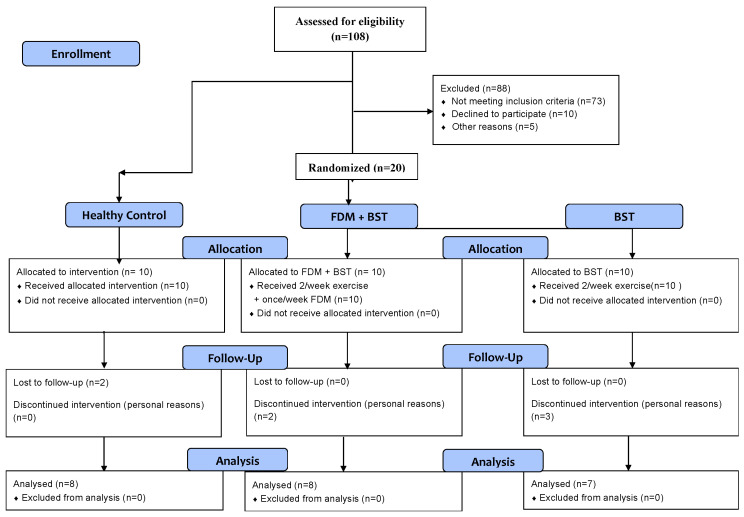
Group allocation and the participant. FDM: Fascial Distortion Model; BST: Balance–Strength Training.

Inclusion criteria [17] were a history of at least one significant ankle sprain, experiencing a feeling of ankle instability or giving way in the last year, not receiving any treatment for the ankle joint in the past year, a score less than 24 on the CAIT scale, and experiencing pain in the ankle joint. The healthy control group were those with no history of an ankle sprain and also, they were excluded if they had any exclusion criteria of the international ankle consortium. Moreover, participants were excluded if they reported the following: a history of previous surgery within the past 18 months to the musculoskeletal structure in either lower extremity; acute injury to musculoskeletal structures of other joints of the lower extremity in the previous 3 months; and any neurological or sensorimotor dysfunction in the lower limb. Group allocation and the participant flow chart are presented in Figure 1.

### 2.2. Outcome Measures

In this study, we conducted various tests to assess the patients’ physical condition before and after the intervention. These pre- and post-test measurements were crucial in evaluating the effectiveness of the intervention and understanding the changes in the patients’ abilities. 

Upon their arrival at the clinic, the participants were informed about the study and provided with a consent form, which they willingly signed. The researchers in charge carried out the measurements, and the process took approximately 30 min to complete for each individual. 

#### 2.2.1. Y-Balance Test Lower Quarter (YBT-LQ)

The YBT-LQ evaluates the dynamic stability, strength, and balance in various directions during a single-limb stance as the opposite leg extends in the anterior, posteromedial, and posterolateral planes. Participants were allowed to have two practice trials per lower extremity without shoes and socks to decrease the external stability of the ankle provided by their shoes [18].

Participants were asked to stand on one leg whilst simultaneously reaching as far as possible with the other leg in three separate directions: anterior, posterolateral, and posteromedial. The subject’s lower limb reach was also normalized to their limb length (from the anterior superior iliac spine to the medial malleolus), which was measured from the anterior superior iliac spine to the distal portion of the medial malleolus. The composite reach distance was calculated by multiplying the sum of the three reach directions by 100, which was then divided by three [19].

#### 2.2.2. Flamingo Balance Test (FBT)

The Flamingo Balance Test is a total-body static balance test and forms a part of the Eurofit Testing Battery. Participants were asked to stand on one leg with their shoes on while standing on a non-slippery wooden beam (50 cm long, 5 cm high, and 3 cm wide) for one minute, with the other leg flexed at the knee and the foot of this leg held close to the buttocks by support of their hand to assess their single-leg dynamic balance. The number of falls from the beam was recorded during one minute of the test. A fall means any loss of balance. If they fell more than 15 times in less than thirty seconds, the test was terminated [20].

#### 2.2.3. Weight-Bearing Lunge Test (WBLT)

Participants were instructed to position their foot so that an imaginary line drawn across the big toe and heel lines up with the tape measure on the ground. In addition, a vertical line parallel to the tape measure was drawn on the wall. Until their knee contacted the wall, participants were told to lunge forward. It was obliged to keep the heel in contact with the floor, while the foot was pushed away from the wall until the knee could only make a little contact with the wall. The maximum distance from the wall to the tip of the big toe was recorded in centimeters (cm) with each centimeter equivalent to about 3.6° of ankle dorsiflexion [21].

#### 2.2.4. Cumberland Ankle Instability Tool (CAIT)

The CAIT [22] is a simple, reliable, and valid questionnaire for discriminating and measuring the severity of functional ankle instability. It is a useful tool for assessing the severity of functional ankle instability, measuring treatment outcome, and monitoring progress. It is a 9-item questionnaire containing a mix of questions related to undamaged, sprained, and unstable ankles. The CAIT score range is from 0 to 30, with higher scores indicating higher stability. The CAIT makes it possible to find, define, and compare more homogeneous subject groups in research. Patients were asked to fill out the questionnaire before and after the intervention. 

#### 2.2.5. Ankle Joint Range of Motion

The range of motion of the ankle joint was measured using goniometry at the beginning and at the end of the procedure. The ankle dorsiflexion, plantarflexion, supination, and pronation were measured [23]. The assessment of plantarflexion and dorsiflexion movements was conducted with the patient comfortably seated with the knee flexed, while the ankle remained in a neutral anatomical stance. This standardized positioning allowed for consistent measurements across all subjects. To precisely quantify the degrees of plantarflexion and dorsiflexion, we employed the lateral malleolus, a prominent bony landmark, as a reference point. This landmark serves as a pivotal indicator of the extent of movement in these particular directions. Furthermore, to ensure accuracy, we established the anterior midline of the talocrural joint as the point of reference for measuring the ROM of these motions.

### 2.3. Interventions

#### 2.3.1. Balance–Strength Training (BST)

One group received two sessions per week of BST for a duration of six weeks. During this period, the exercises applied were progressive. Each session started with a 5 min warm-up and lasted about 45 min, followed by 5 min of stretching exercises at the end. In the first week, patients started with resistance and bodyweight training with a focus on the lower leg muscles, ankle mobility, and dynamic balance. By week six, patients experienced dynamic and static balance, jumping and landing exercises, harder resistance exercises, and complex exercises.

The ankle–foot complex is typically the only part of the body in contact with the ground during most activities; ankle proprioception may be one of the more crucial factors influencing balance control [24]. Then, during and/or after the strengthening program, interventions should aim to re-program neuromuscular control during landing/cutting by optimally distributing ground impact across the ankle, knee, and hip joints. And restoring dorsiflexion ROM through calf muscle stretching and/or joint mobilization techniques is needed for injury prevention in this population [25].

#### 2.3.2. FDM Manual Therapy

The other treatment that was applied on the affected ankle of the CAI individuals in this research for the second group of the CAI patients, in addition to the BST, follows the concept of FDM, which is described by Dr. Typaldos [26]. FDM is a medical concept with its own diagnosis and as a concept, it remains neutral in method. FDM itself is not a manual treatment; rather, it involves specific manual actions aimed at addressing Fascial Distortions. When these actions are employed, it is referred to as FDM manual treatment. Nevertheless, this method represents only a portion of the comprehensive perspective and concept of FDM. In the context of FDM, medical conditions such as an ankle sprain or ankle instability are regarded as one or more out of six Fascial Distortions. These Fascial Distortions refer to various alterations in the connective tissue, which explain patients’ complaints. Once diagnosed, these specific alterations necessitate certain interventions aimed at correcting them. When these corrections are made through manual techniques performed by the therapist, the method is known as FDM manual therapy.

During the six-week intervention, one group of patients received this treatment once a week along with their regular exercises that took about 30 min for everyone. Out of six common distortions that were introduced in this method, four distortions were found during the physical examination that focused on the patient’s body language, clinical testing, and history taking before each session. These four distortions that were found and manually corrected are trigger band (TB), continuum distortion (CD), folding distortions (refolding (rFD) and unfolding (uFD)), and cylinder distortion (cyD) [26].

**Trigger band (TB):** the most common distortion patients tend to unconsciously move their fingers in a sweeping manner along a certain pathway while explaining their discomfort. The solution to this issue is to straighten the twisted fibers and smooth out the wrinkles with the therapist’s thumb. It is also the only distortion that can become chronic if adhesions between the distorted fibers are built. Therefore, it is frequently encountered in CAI patients with loss of balance and muscle weakness combined with pressure pain that is characterized by pulling and burning sensations. In addition, in the case of chronic TBs, since they are held together by adhesions, they sometimes require more treatments and more force in order to correct them.

**Continuum distortion (CD):** These are minor injuries at the bone–ligament transition zone and patients usually indicate the area by pointing with their finger. The ROM is also restricted to one plane in the affected joint. The approach to treatment is to realign the bone components by firm and continuous pressure into the distortion to shift the transition zone.

**Folding distortions (rFD and uFD):** These are three-dimensional changes of the fascia and are reported as deep pain in the joint while moving under stress or pressure in uFD or even without load in the case of rFD. The treatment approach is traction and torquing the torqued fascia or compression of the joint, respectively.

**Cylinder distortion (cyD):** These types of distortions result in deep and strange pain in non-jointed areas and are sometimes referred to as jumping pain from one area to another. Patients often show a sweeping motion with their palm along a wide area, squeezing, pinching, or messaging. There are a variety of techniques to correct this distortion such as the double thumb technique, Indian burn, the squeegee technique, and pinching. It is important to note that some techniques may have to be repeated in different regions surrounding the ankle joint to fully repattern the distorted fascia.

### 2.4. Statistical Analyses

In this study, IBM SPSS Statistics 28.0 was used to analyze data from study participants. Prior to analysis, we confirmed data normality through the Shapiro–Wilk test. In all statistical analyses, a significance level of *p* < 0.05 was employed to identify statistically significant differences.

Descriptive statistics, including means and standard deviations, were employed to summarize the demographic and baseline characteristics of the study population. Group comparisons were conducted using independent-sample *t*-tests to assess potential differences among the three groups (FDM + BST, BST, and control). Additionally, paired-sample *t*-tests were used to compare each group’s improvement at the post-test with their pre-test results, and Cohen’s effect size (Cohen’s d) was computed to provide an estimate of the practical significance of the observed differences.

In this study, changes from the pre-test to the post-test were measured using a repeated-measure analysis of variance (rmANOVA). Group distinctions were examined as the between-subject factor, while changes over time served as the within-subject factor. To address any deviations from sphericity, Greenhouse–Geisser probability levels were employed as needed. Additionally, we conducted post hoc analyses and applied a Bonferroni correction for multiple comparisons. Alongside the *p*-values, we also reported effect sizes using partial eta-squared (ηp^2^).

## 3. Results

Table 1 compares the general characteristics of the three groups, in addition to the baseline measurements. There were no significant differences between age, weight, height, and BMI between groups.

The results presented in Table 1 provide a snapshot of the demographic and baseline data for participants in three different groups: FDM + BST, BST, and control. No significant differences are observed for age, height, weight, and BMI of the participants (*p* = 0.394, 0.650, 0.723, and 0.862; effect size = 0.09, 0.04, 0.03, and 0.570).

Table 2 and Table 3 report the statistical analysis for ankle ROM, WBLT, FBT, YBT-LQ, and CAIT score within groups. A rmANOVA was conducted to evaluate the null hypothesis for changes in subjects’ ankle ROMs, WBLT, FBT, YBT-LQ, and CAIT score when measured at pre-test and post-test. Mauchly’s test indicated that the assumption of sphericity had been violated, therefore degrees of freedom were corrected using Greenhouse–Geisser.

The Group Effect (*p* < 0.001, ηp^2^ = 0.539 for dorsiflexion, *p* = 0.778, ηp^2^ = 0.025 for plantarflexion, *p* = 0.682, ηp^2^ = 0.037 for pronation, *p* = 0.828, ηp^2^ = 0.019 for supination) shows significant differences among the three groups at pre-test only for the dorsiflexion ROM. The Time Effect (*p* = 0.088, ηp^2^ = 0.139 for dorsiflexion, *p* < 0.001, ηp^2^ = 0.638 for plantarflexion, *p* = 0.001, ηp^2^ = 0.461 for pronation, *p* < 0.001, ηp^2^ = 0.615 for supination) suggests a significant change in the ankle joint ROM except dorsiflexion ROM over time within all groups.

The Group Effect (0.010, ηp^2^ = 0.367 for WBLT, *p* = 0.255, ηp^2^ = 0.127 for FBT, *p* = 0.030, ηp^2^ = 0.297 for YBT-LQ, *p* < 0.001, ηp^2^ = 0.734 for CAIT score) shows significant differences among the three groups at post-test for WBLT and CAIT score. The Time Effect (0.001, ηp^2^ = 0.460 for WBLT, *p* < 0.001, ηp^2^ = 0.353 for FBT, *p* < 0.001, ηp^2^ = 0.531 for YBT-LQ, *p* < 0.001, ηp^2^ = 0.832 for CAIT score) suggests a significant change in all tests and CAIT score over time within all groups.

Pairwise comparisons have indicated significant differences between the FDM + BST and BST groups for supination ROM (0.008) and similarly for WBLT, FBT, YBT-LQ, and CAIT score (0.041, 0.40, 0.023, and 0.008).

Paired-sample *t*-test results were used to compare each group’s performance at the post-test, compared to their pre-test results. For the FBT and YBT-LQ, the FDM + BST group significantly improved (*p* = 0.015, effect size = 1.12 for FBT and *p* = 0.004, effect size = −1.49 for YBT-LQ), while the BST group showed non-significant changes (*p* = 0.289, effect size = −0.44 for FBT and *p* = 0.058, effect size = −0.88 for YBT-LQ). For the WBLT, there was a significant increase in the FDM + BST group (*p* = 0.007, effect size = −1.34). The BST group also showed a significant improvement (*p* = 0.046, effect size = −0.95). The CAIT scores increased significantly for both the FDM + BST and BST groups (*p* = < 0.001 and 0.003; effect size = −3.28 and −1.78, respectively), indicating a reduction in ankle instability. However, the control group’s CAIT score remained unaltered as they were the healthy control group.

In terms of ankle joint ROM, FDM + BST showed improvements in plantarflexion, pronation, and supination ROM (*p*: 0.010, 0.020, <0.004; effect sizes: −1.25, −1.06, −2.39). On the other hand, the BST group showed significant improvements only for plantarflexion and pronation ROM (*p*: 0.002 and 0.035; effect size = −2.06 and −1.02). However, no significant difference was observed for dorsiflexion ROM in both groups (*p* = 0.57, 0.23; effect sizes: −0.5, 0.21).

## 4. Discussion

Recovering from ankle sprains can be challenging since the reason for their recurrence has not been definitively identified, even though researchers have extensively studied and investigated them [27]. Despite the availability of various manual therapy techniques, there exists a notable challenge in achieving successful rehabilitation following an acute ankle sprain, often resulting in the development of CAI. Ankle injuries are inversely correlated with both balance control and ankle proprioception and have a substantial impact on one’s ability to balance [24]. Additionally, it was reported by Brandolini and colleagues that fascia manipulation has the potential to effectively prevent ankle injuries throughout the entire football season. The long-term benefits were observed even six months after just three 45 min treatment sessions [28]. Therefore, the primary goal of this clinical trial is to investigate the efficacy of integrating FDM concepts with BST in enhancing static and dynamic balance as well as ankle joint ROM.

Recently, a novel method to overcome ankle sprain, ref. [29], revealed that early fascia manipulation improves joint mobility. It may also reduce the delay of tissue healing and, thus, optimize further rehabilitation of the sprained ankle and any injury to the fascia structure; symptoms like weakness and range of motion limitations are expected to happen [16]. The results suggest that the fascia distortion model method is a new intervention that could be used for subjects with chronic ankle instability. This method has the potential to effectively enhance proprioception and dynamic balance [16]. It is worth noting that myofibroblasts play an active role in regulating the tension of myofascial tissue, which can significantly impact the dynamics of the musculoskeletal system [30]. In addition, in an ultrasound imaging study, it was found that the epimysial fascia is thicker in basketball players with a previous ankle sprain compared to those without such a history [31]. 

It is important to highlight that this study presents results related to the affected ankle. However, exercises were applied to both CAI groups on both legs due to reports by Hertel and Deodato [32,33] that unilateral CAI also impairs motor control and muscle function on the contralateral side and in the long term, if CAI persists, it may lead to joint-degenerative pathologies, such as osteoarthritis [34]. Moreover, when surgery is not a preferred management method to treat unilateral CAI, rehabilitation protocols should focus on both sides [35].

### 4.1. Dynamic and Static Balance

Contrary to our expectations, the FDM + BST group exhibited significantly better outcomes in the FBT and YBT-LQ compared to the BST group. This unexpected finding may be attributed to the correction of several trigger bands during the weekly FDM manual therapy sessions. According to Dr. Typaldos [26], balance disturbances result from any distortion to trigger bands, and correcting them can improve both static and dynamic balance. Our study supports the hypothesis that the FDM + BST group demonstrated a better performance in the Flamingo Balance Test. Further details about the study design, intervention, and outcomes are provided in subsequent sections. To date, no study has assessed static balance on individuals with CAI using the FBT on any therapeutic approach, and our findings suggest that the combination of FDM concepts and BST can result in better performance during the FBT. In addition, previous studies have shown that individuals with CAI have lower scores on the YBT-LQ compared to healthy individuals [36,37]. While previous studies [36,37] have focused mainly on the anteromedial, posteromedial, and posterolateral directions, one study [19] suggested that calculating the composite reach distance (the mean of three reach directions) reduces measurement error and increases interrater test–retest reliability.

Our findings are in line with the improvement observed in both groups when compared to their pre-test performances. Notably, there was a significant difference in the FDM + BST group. This aligns with the results of another study, which indicated that ankle taping and bandaging effectively increased the composite reach distance in the YBT-LQ after 2 weeks and 2 months [38]. Furthermore, it was suggested that individuals with CAI who underwent rehabilitation could reach further in the posteromedial, posterolateral, and lateral directions of the Star excursion balance test compared to CAI controls. However, no improvement was observed in the composite reach distance in either the resistance-band protocol or proprioceptive neuromuscular facilitation (PNF) group [39]. This study also mentioned that in their research, they exclusively used strengthening training, which may account for the discrepancy between their findings and the current literature, as well as our own study, where CAI individuals who received either balance or balance–strength training exhibited significant improvements in the YBT-LQ compared to their pre-test performance.

### 4.2. Ankle Joint Mobility

To develop effective rehabilitation plans targeting ankle dorsiflexion ROM and functional movement deficiencies, the WBLT is a reliable measure that exhibits a high correlation with standard dorsiflexion ROM assessments [40].

An earlier study discovered that a combination of mechanical and functional impairments that affected the health-related quality of life of people who had been diagnosed with CAI was significantly impacted by dorsiflexion ROM as determined by the WBLT [41]. In a separate study in 2016 [42], the joint mobilization group demonstrated superior WBLT results compared to the control group. In our study, both the FDM + BST and BST groups showed significant post-test improvements compared to their pre-test results, with the FDM + BST group exhibiting a higher rate of improvement.

Furthermore, another study [43] highlighted the benefits of joint mobilization techniques for CAI patients in increasing dorsiflexion ROM. Marrón-Gómez et al. [44] observed that mobilization with movement (MWM) or the high-velocity and low-amplitude (HVLA) manual technique had a lasting effect on increasing joint mobility for at least two days in the CAI population. Similarly, it is accepted that myofascial techniques in addition to strength training can be beneficial to increase ankle ROM, strength, and stability in footballers with recurrent ankle sprains, and its addition to Kinesiotaping can have a positive impact on ankle mobility and strength [45]. In our study, as part of the Fascial Distortion Model (FDM) concept, patients received HVLA manipulation based on their diagnostic examinations and discovered distortion, resulting in higher post-test effects, particularly when combined with BST, which aligns with the result of the study by Kamani in 2021, where a significant active dorsiflexion ROM was achieved after fascia manipulation (FM) [46]. Another study by Cruz-Díaz in 2020 demonstrated that adding self-mobilization to a CrossFit intervention outperformed CrossFit training alone in improving ankle dorsiflexion ROM when assessed by WBLT [47].

Some reports found no significant differences in active ankle joint ROM compared to control groups [48] and in another study [49], ankle joint range of motion in all directions did not change significantly while comparing the pre-test and post-test results. However, in the current study, FDM + BST showed improvements in plantarflexion, pronation, and supination ROM, while the BST group showed significant improvements only for plantarflexion and pronation ROM. However, no significant difference was observed for dorsiflexion ROM in both groups. Furthermore, in the case of improvement between groups, the FDM + BST group showed a significantly better improvement in supination ROM than the BST group.

### 4.3. Patients’ Reported Outcomes

The CAIT is a simple, reliable, and valid questionnaire for discriminating and measuring the severity of functional ankle instability. It proves to be an essential tool for assessing the severity of this condition in order to measure treatment outcomes and track progress [22]. An increase in CAIT scores has been reported for both the FDM + BST and BST groups, indicating a reduction in ankle instability.

Several studies have employed the CAIT score in order to assess the patient-reported outcomes after interventions, consistently revealing significant differences compared to baseline results [43,50,51]. In one study by Kim and colleagues [50], a combined regimen of strength and proprioceptive training led to an impressive average CAIT score improvement of 5.3 points, surpassing the 3.2-point increase observed with strength training alone. This outcome highlights the effectiveness of combined muscle strengthening and proprioceptive exercises over muscle strengthening exercises alone. Cruz-Diaz [52] documented a 3.8-point rise following a six-week balance training program. In a study by Wang in 2022 [51], an impressive 8.92-point increase in CAIT scores was reported after isokinetic strength training. The CAIT increases observed in the current study (8.12 with FDM + BST and 4.58 with BST) closely resemble the outcomes of earlier studies. Therefore, both of our interventions were just as effective as other established protocols in reducing instability as assessed by the CAIT. 

## 5. Limitations

Our study was subject to certain limitations that should be acknowledged. Firstly, the presence of a language barrier posed a challenge, resulting in a limited number of patients included in the study. Additionally, there was a lack of specific measurements prior to advancing the exercises in both groups, which may have affected the accuracy of our findings. Furthermore, we did not differentiate between subjects with mechanical and functional instabilities within the chronic ankle instability (CAI) cohort, which could have provided valuable insights into the effectiveness of the interventions for each subgroup. It was also proven that the menstrual cycle modulates ankle ligament laxity. However, this study encompassed both male and female participants as it was not feasible to incorporate only males into the study due to difficulties in securing the subjects.

## 6. Conclusions

To the best of our knowledge, this study represents the first attempt to compare the effectiveness of combining FDM manual therapy with a rehabilitation program versus using only a rehabilitation program in patients with CAI. While the post-test results indicated significant improvement across all measured functional tasks and ankle ROMs, our findings indicate that the combined protocol of FDM + BST is particularly advantageous for enhancing performance in the WBLT, FBT, YBT-LQ, and supination ROM, as well as the CAIT score.

## Figures and Tables

**Table 1:** Participants’ demographic and baseline data by groups.

	Mean ± SD			*p* (Effect Size)
Groups	FDM + BST	BST	Control	
Sample Size	8	7	8	
Age	21.37 ± 3.24	20.43 ± 1.90	22.37 ± 2.66	0.394 (0.09)
Height (cm)	173.5 ± 9.24	169.86 ± 9.61	174.87 ± 12.49	0.650 (0.04)
Weight (kg)	68.5 ± 12.5	66.14 ± 11.24	71.25 ± 12.61	0.723 (0.03)
BMI (kg/m^2^)	22.79 ± 3.93	22.31 ± 2.71	23.23 ± 2.90	0.862 (0.57)
Number of Sprains	3.50 ± 1.69	4.29 ± 2.62	0.00	<0.001 (0.01)
CAIT	14.37 ± 14.38	19.71 ± 3.90	30.00	<0.001 (0.76)

Body Mass Index (BMI); Cumberland Ankle Instability Tool (CAIT). The mean difference is significant at 0.05 level.

**Table 2:** Pre-test and post-test comparison of ankle ROM.

		Participants			rmANOVA		
		FDM + BST	BST	Control	Group Effect *p* (ηp^2^)	Time Effect *p* (ηp^2^)	Time × Group *p* (ηp^2^)
Dorsiflexion (°)	pre-test	12.13 ± 7.08	16.43 ± 9.14	21.13 ± 4.22	<0.001 (0.539)	0.088 (0.139)	0.542 (0.059)
	post-test	10.63 ± 0.92	11.43 ± 1.40	19.75 ± 3.58			
Plantarflexion (°)	pre-test	48.13 ± 17.08	46.14 ± 10.29	58.13 ± 6.47	0.778 (0.025)	<0.001 (0.638)	0.003 (0.449)
	post-test	66.63 ± 4.72 *	65.00 ± 4.44 *	58.75 ± 5.92			
Pronation (°)	pre-test	16.63 ± 8.21	15.57 ± 5.88	22.38 ± 4.90	0.682 (0.037)	0.001 (0.461)	0.029 (0.298)
	post-test	25.38 ± 8.50 *	23.57 ± 8.73 *	22.50 ± 5.24			
Supination (°)	pre-test	30.38 ± 9.53	31.57 ± 10.41	36.38 ± 6.68	0.828 (0.019)	<0.001 (0.615)	<0.001 (0.602)
	post-test	43.63 ± 9.86 *	37.14 ± 6.91	35.63 ± 8.16			

The mean difference is significant at 0.05 level. * Significant improvement of the group at post-test compared to their pre-test results.

**Table 3:** Pre-test and post-test comparison of WBLT, FBT, YBT-LQ, and CAIT score.

		Participants			rmANOVA		
		FDM + BST	BST	Control	Group Effect *p* (ηp^2^)	Time Effect *p* (ηp^2^)	Time × Group *p* (ηp^2^)
WBLT (cm)	pre-test	6.25 ± 2.25	8.93 ± 4.55	12.50 ± 2.83	0.010 (0.367)	0.001 (0.460)	0.009 (0.376)
	post-test	9.25 ± 2.72 *	9.57 ± 2.76 *	13.00 ± 3.25			
FBT	pre-test	6.00 ± 4.34	3.14 ± 2.12	1.63 ± 3.11	0.255 (0.127)	<0.001 (0.353)	0.008 (0.379)
	post-test	1.63 ± 1.85 *	2.29 ± 1.89	1.50 ± 3.21			
YBT-LQ (%)	pre-test	71.92 ± 7.96	80.02 ± 6.00	84.17 ± 5.99	0.030 (0.297)	<0.001 (0.531)	0.026 (0.305)
	post-test	84.56 ± 4.10 *	89.59 ± 7.85	85.49 ± 6.49			
CAIT	pre-test	14.38 ± 5.66	19.71 ± 3.90	30.00 ± 0.00	<0.001 (0.734)	<0.001 (0.832)	<0.001 (0.763)
	post-test	22.50 ± 3.34 *	24.29 ± 2.98 *	30.00 ± 0.00			

Weight-Bearing Lunge Test (WBLT); Flamingo Balance Test (FBT); Y-Balance Test Lower Quarter (YBT-LQ); Cumberland Ankle Instability Tool (CAIT). The mean difference is significant at 0.05 level. * Significant improvement of the group at post-test compared to their pre-test results.

## Data Availability

The data presented in this study are available on request from the corresponding author (Dr. Péter Sándor Tardi).
